# Treatment of Hypogonadism: Current and Future Therapies

**DOI:** 10.12688/f1000research.10102.1

**Published:** 2017-01-23

**Authors:** Arthi Thirumalai, Kathryn E. Berkseth, John K. Amory

**Affiliations:** 1 Division of Metabolism, Endocrinology, and Nutrition, Department of Medicine, University of Washington, Seattle, WA, USA; 2 Center for Research in Reproduction and Contraception, Department of Medicine, University of Washington, Seattle, WA, USA

**Keywords:** hypogonadism, testosterone therapy, dihydrotestosterone gel, selective androgen receptor modulator, aromatase inhibitor

## Abstract

The treatment of hypogonadism in men is of great interest to both patients and providers. There are a number of testosterone formulations currently available and several additional formulations under development. In addition, there are some lesser-used alternative therapies for the management of male hypogonadism, which may have advantages for certain patient groups. The future of hypogonadism therapy may lie in the development of selective androgen receptor modulators that allow the benefits of androgens whilst minimizing unwanted side effects.

## Introduction

The Endocrine Society Clinical Practice Guideline defines male hypogonadism as “a clinical syndrome that results from failure of the testes to produce physiological levels of testosterone due to disruption of one or more levels of the hypothalamic-pituitary-testicular axis” ^1^. Given that testosterone concentrations decline with age ^2, 3^ and the increasing awareness of the signs and symptoms of hypogonadism, prescriptions for testosterone therapy have increased significantly in the last several years ^4^.

In this review, we will briefly summarize the risks and benefits of testosterone replacement therapy. In addition, we will describe the existing options for testosterone therapy, including several newer formulations of testosterone under development. Lastly, we will discuss the data available on alternative therapies occasionally used for the treatment of male hypogonadism.

## Risks and benefits of testosterone therapy

For men with symptomatic hypogonadism, there are a number of potential clinical benefits with testosterone replacement therapy, including improvements in libido, erectile function, muscle strength and body composition (including decreased fat mass, increased lean mass, and improved bone mineral density), mood, and cognition ^1^. The potential clinical benefits of testosterone therapy must be carefully weighed against potential risks. Potential adverse effects of testosterone replacement include erythrocytosis, increases in prostate-specific antigen (PSA) and worsening of prostate disorders (including benign prostatic hyperplasia [BPH]), dermatologic effects, including acne and skin irritation, and worsening of existing obstructive sleep apnea ^1^. In addition, exogenous testosterone administration leads to the suppression of luteinizing hormone (LH), decreased intra-testicular testosterone concentrations, and reduced spermatogenesis ^5^. Thus, testosterone replacement therapy is not appropriate for hypogonadal men desiring fertility.

Notably, the US Food and Drug Administration (FDA) has recently added important warnings to testosterone products. The first is a warning added to all testosterone preparations highlighting a potential increased risk of cardiovascular disease (including myocardial infarction and stroke) in patients taking testosterone, although additional studies are needed to guide clinicians in better understanding these potential risks, as the magnitude of these risks are unknown. However, until additional information becomes available, the current recommendation is that clinicians in practice should discuss these potential risks with all patients when starting or continuing testosterone replacement therapy ^6, 7^.

In October 2016, the FDA added additional warnings to the labeling of testosterone products to alert prescribers to the potential for abuse of testosterone and other anabolic androgenic steroids. The FDA statement outlined potential adverse effects of abuse of anabolic androgenic steroids, including myocardial infarction, heart failure, stroke, liver injury, male infertility, and mood changes including depression, aggression, and hostility. They also raised concerns that abuse of high doses of testosterone was reported to be associated with potential withdrawal symptoms including depression, irritability, fatigue, insomnia, and decreased libido. The potential for abuse of testosterone and other androgens should be considered in both adult and adolescent populations.

## Testosterone replacement therapy

There are a variety of testosterone preparations currently on the market in the US ( Table 1) and additional formulations available abroad and in development (see below). In choosing the testosterone formulation for an individual patient, clinicians must consider the individual values and preferences of the patient as well as other factors including cost, convenience, and availability. Owing to their ease of use and relatively low cost, injectable and transdermal testosterone preparations are currently the most widely used in the US.

**Table 1.** Testosterone preparations.

Formulation	Preparation (US trade name)	Dosage Forms	Usual Dosing*	Site of Application	Advantages	Disadvantages and Risks	Approximate Cost per Month**
**Intramuscular**							
Intermediate acting	Testosterone cypionate (Depo- testosterone)	100 mg/mL or 200 mg/mL	100–200 mg every 2 weeks or 50–100 mg every 1 week	Thigh or buttock	Home IM injection, infrequent treatment, low cost, high efficacy	Peak effects/fluctuating testosterone levels, pain/irritation at injection site	$15–60 (generic) $50–70 (brand)
	Testosterone enanthate (Delatestryl)	200 mg/mL				$15–35 (generic) $45–50 (brand)
Long acting	Testosterone undecanoate (Aveed)	250 mg/mL	750 mg initially, then 750 mg at 4 weeks, then 750 mg every 10 weeks ongoing	Buttock	Long acting	Administered in office/hospital by REMS-certified provider, risk of pulmonary oil microembolism and anaphylaxis	$1050 (plus cost of injection)
**Transdermal**							
Gels	AndroGel (1% gel)	25 mg in 2.5 g packet or 50 mg in 5 g packet	50–100 mg daily	Dry intact skin or back, abdomen, upper thighs, or arm	Steady serum testosterone concentration	Risk of transfer, requires daily application, may not achieve normal T levels in all men, occasional skin irritation	$175–400 (generic) $500–525 (brand)
	Testim (1% gel)	50 mg in 5 gm packet					$160–320 (generic) $480–520 (brand)
	AndroGel (1.62% gel)	20.25 mg in 1.25 g packet, 40.5 mg in 2.5 g packet, 20.25 mg per actuation, metered dose pump	20.25–81 mg daily				$480–550 (brand only)
	Fortesta (2% gel)	10 mg per actuation, metered dose pump	10–70 mg daily	Dry intact skin of front and inner thighs	Ease of application		$160–400
	Axiron (2% solution)	30 mg per actuation, metered-dose pump	30–120 mg daily	Dry, intact skin of axilla	Ease of application, reduced risk for transfer		$260–1,200
Patch	Androderm	2 mg/24 hour patch 4mg/24 hour patch	2–6 mg daily	Dry intact skin of arm or torso	Limited risk of transfer, no injection	Skin irritation/rash (about one- third of men), daily application	$475–510
**Other**							
Implanted Subcutaneous Pellet	Testopel	75 mg pellets	150–450 mg every 3–6 months	Implanted into subcutaneous fat of buttock, lower abdominal wall, or thigh	No risk of transfer, no daily treatment	Extrusion, infection, fibrosis at pellet sites. Placed in clinic/ hospital by trained provider under sterile conditions.	$150–175 (plus cost of pellet placement) cost estimate based on dose 150 mg every 3 months
Nasal	Natesto	5.5 mg per actuation, metered dose pump applicator	11 mg (two pumps, one in each nostril) three times daily	Intranasal	Minimal risk of transfer	Frequent administration, rhinorrhea, epistaxis, sinusitis, nasal scab	$600–700
Buccal	Striant SR	30 mg buccal system	30 mg twice daily	Adhere to depression in the gingiva superior to upper incisors	No injection	Frequent administration, gingival irritation	$550–600
Oral (testosterone undecanoate)	Andriol	40 mg	40–80 mg orally, three times daily	Oral, taken with fat-containing meal	No injection	Frequent dosing required, relatively low testosterone delivery	$200–$300
Oral (alkylated)	Not recommended						

*Usual doses are listed but dosing should be adjusted based on specific patient factors and clinician judgment. **Cost data based on average cost purchasing monthly supply, various suppliers as listed on goodrx.com at the time of publication and estimated costs at University of Washington Medical Center for facility-administered testosterone undecanoate and Testopel. IM, intramuscular; REMS, Risk Evaluation and Mitigation Strategy; T, testosterone. Table adapted with permission from 44.

## Injectable formulations

Two intermediate-acting injectable testosterone formulations are currently available in the US market: testosterone enanthate and testosterone cypionate ^8, 9^. These are usually dosed every 1–2 weeks. In contrast, a newer long-acting testosterone ester, testosterone undecanoate (“Nebido” in Europe and “Aveed” in the US), can be dosed every 6–12 weeks. All of these formulations are administered as intramuscular injections. The majority of patients are able to administer injections independently at home with the help of a partner. Intramuscular testosterone formulations are highly effective in improving symptoms of hypogonadism. Additional benefits of long-acting injectable preparations include low cost of therapy (as compared to other preparations) and they more reliably achieve therapeutic serum concentrations of testosterone (as compared to transdermal preparations), thus reducing the need for routine monitoring of serum testosterone concentrations during therapy. With testosterone enanthate and testosterone cypionate, testosterone concentration and clinical effects peak 1–2 days after the injection and wane over the subsequent 2 weeks ^10^. For some patients, the fluctuations in serum testosterone concentrations can lead to adverse impacts on mood, energy, and sexual function, which can be bothersome or disruptive. In these cases, alternate dosing using half the usual dose administered weekly (instead of full dose every 2 weeks) or use of an alternate testosterone preparation may be preferred.

In contrast, testosterone undecanoate achieves relatively stable testosterone concentrations. Unfortunately, the relative large volume of testosterone undecanoate injections can be associated with the risk of pulmonary oil microembolism and anaphylaxis, albeit rarely. As a result, testosterone undecanoate is available only through a Risk Evaluation and Mitigation Strategy (REMS) program and must be administered by a trained, registered care provider in an office or hospital setting – it cannot be self-administered at home by the patient.

## Transdermal formulations

Transdermal testosterone gels are widely available and popular among both patients and clinicians. There are multiple transdermal gel formulations currently available in the US including AndroGel, Testim, Fortesta, and Axiron ( Table 1). They are supplied in sachets, tubes, or metered-dose pumps and are applied by hand to the skin of the arms, torso, or thighs. Newer concentrated testosterone preparations offer the advantage of applying a smaller volume of gel at each dose. Benefits of transdermal gels include high efficacy for the management of symptoms of hypogonadism, ease of home administration, and minimizing fluctuations in testosterone concentration from day to day. As a result, these formulations may be preferable for patients who struggle with peak and trough effects associated with the use of intermediate-term intramuscular injections. Risks of transdermal gels include mild skin irritation and potential for skin-to-skin transfer to others. All patients using gels should be instructed on careful hand washing after gel application and avoiding skin-to-skin contact with others (particularly female partners or children) on the gel-treated areas ^11^. In contrast to injectable testosterone preparations, absorption of transdermal testosterone gel can be quite variable. If a patient using a transdermal formulation of testosterone has no improvement in symptoms, it is reasonable to measure the serum testosterone concentration and adjust the dose as needed to achieve adequate circulating testosterone concentrations.

When the risk of skin-to-skin transfer of testosterone is of concern, the use of a transdermal patch may be appropriate. There is currently one transdermal testosterone patch preparation (Androderm) available in the US. Use of the patch has been limited by relatively high rates of skin irritation, with up to one-third of men who use the patch experiencing significant skin irritation. Similar to other transdermal testosterone preparations, monitoring of circulating serum testosterone concentration and appropriate dose adjustment are reasonable with transdermal patches, since testosterone absorption can be variable.

## Other testosterone formulations

A subcutaneous testosterone pellet (Testopel) is available for the treatment of hypogonadism ^12^. Testopel is placed in the subcutaneous fat of the buttock, lower abdomen, or thigh every 3–6 months. Pellets are placed using sterile technique in an office or hospital setting and cannot be injected by the patient at home. Risks with subcutaneous testosterone pellets include infection, fibrosis, and pellet extrusion. Benefits include eliminating risk of skin-to-skin transfer, infrequent dosing, and relatively stable serum testosterone concentrations.

Nasal and buccal testosterone preparations are also available for the treatment of hypogonadism and may be useful in limited clinical settings where other testosterone preparations are not effective or appropriate. Use of nasal and buccal preparations is limited because of the potential for nasal/sinus and gingival irritation, limited published data on use of the nasal preparation, and animal studies suggesting possible increases in central nervous system testosterone levels above that expected with other formulations ^13, 14^.

Oral testosterone undecanoate (Andriol) is available in many countries outside the US; however, serum testosterone concentrations achieved with this formulation can be low and administration must occur with a fat-containing meal. New self-emulsifying drug formulations of testosterone undecanoate are under development and may reach the market in the next 1–2 years ^15^. However, high post-dose serum peaks and a relatively large degree of inter-individual variability in achieved drug concentrations are issues for these formulations. In response, the companies developing these newer formulations of testosterone undecanoate are developing dosing algorithms to ensure patients receive the appropriate dose to achieve therapeutic concentrations of testosterone. Other oral preparations, especially those alkylated at the 17-carbon position, such as methyltestosterone, are associated with hepatotoxicity and are not recommended for use.

Some newer testosterone formulations have recently come to the global market. These include both transdermal and injectable formulations. One of the transdermal formulations includes a hydro-alcoholic 2.5% testosterone gel (Testocur) that is approved for use in Germany. This drug was studied after application both transdermally and transscrotally, and compared with Androderm 2.5% patches applied transdermally in an open-label, parallel group, randomized controlled fashion in previously treated hypogonadal men for 24 weeks ^16^. While the scrotal gel and patches achieved equivalent serum androgen (testosterone and dihydrotestosterone [DHT]) concentrations, the transdermal gel actually achieved higher concentrations. The transdermal gel was also better tolerated than the patches whilst proving equivalent in safety outcomes such as hematocrit, serum PSA, and prostate volumes. Another formulation that is currently approved for use in Australia is an alcohol-free testosterone cream (AndroForte 5, 50 mg/mL or 5%, Lawley Pharmaceuticals) ^17^. This was studied in an open-label, randomized crossover study, comparing it with 1% testosterone gel in hypogonadal men with a treatment period of 30 days. Pharmacokinetic end points of both were comparable and there were no differences in serum hormone concentrations.

## Alternative therapies for treatment of male hypogonadism

Given the concerns surrounding the safety and benefits of testosterone therapy in men, other agents have also been used for the treatment of male hypogonadism. In particular, in younger men who are interested in fertility, testosterone replacement therapy is not recommended.

### Dihydrotestosterone gel

Testosterone is metabolized by 5α-reductase to DHT in the body. DHT has a stronger affinity than does testosterone for the androgen receptor in certain tissues such as the prostate, skin, and external genitalia. DHT gel is used for the treatment of hypogonadism in France and Belgium. The theoretical advantages of using DHT gel over testosterone in men would include mainly that (a) its use does not translate into increased intra-prostatic DHT concentrations and hence the risk of adverse prostate outcomes is lower ^18^, and (b) it is also not aromatized and hence gynecomastia is not a concern. Studies of DHT gel have also shown (like testosterone replacement) improved sexual function and muscle mass, lower fat mass ^19^, and favorable effects on lipid profiles ^20^. The downside to using DHT gel, however, includes the higher cost and the theoretical harm of lack of aromatization to estradiol (E2), which could impact bone mineral density and libido. Additionally, it is uncertain if the supra-physiologic serum DHT concentrations that are achieved with DHT gel ^18^ can have long-term unfavorable effects of their own.

### Human chorionic gonadotropin

In a healthy male, pulsatile gonadotropin-releasing hormone (GnRH) secretion stimulates follicle-stimulating hormone (FSH) and LH secretion from the pituitary, which in turn act on the Sertoli and Leydig cells, respectively, and support spermatogenesis and intra-testicular testosterone production ^21^. In hypogonadal men, the level to which spermatogenesis is impaired depends on both the etiology of hypogonadism and the time of onset (pre-pubertal versus post-pubertal), since that affects the baseline testicular volume and the number and functionality of germ cells. Human chorionic gonadotropin (hCG) therapy (recombinant hCG) is traditionally used in hypogonadal men desiring fertility, since it shares a receptor with LH and produces similar effects. The dose is usually titrated to a serum testosterone concentration in the mid-normal range. After 6 months of therapy, sperm concentrations are assessed and if no response is noted, then FSH therapy, either human menopausal gonadotropin (hMG) or recombinant human FSH (rhFSH), is added. Response times can be as long as 1–2 years for the combination, and success is higher in men with testicular volumes >8 cc and later onset of hypogonadism ^22^. Studies have also looked at the role of hCG therapy in the treatment of hypogonadism in men without fertility concerns, and it has been shown to improve hypogonadal symptoms ^23^ and have favorable effects on body composition (increased fat-free mass and lower fat mass), lipid profile (lower total cholesterol, low-density lipoprotein cholesterol, and triglycerides) ^24^ and bone formation ^25^. hCG therapy also seems to be less likely to cause the adverse effects that testosterone is associated with in terms of prostate health, hematocrit, sleep apnea, and gynecomastia; however, it entails more frequent (2–3 times a week) injections than does testosterone and can also cause testicular enlargement ^26^.

### Clomiphene citrate

Clomiphene is a selective estrogen receptor modulator that has weak anti-estrogen action. It competes with estrogen for binding at the estrogen receptor and increases GnRH secretion, thereby increasing LH secretion ^27^ and consequently serum testosterone concentrations. For this reason, it works only in individuals with hypogonadotropic hypogonadism with an otherwise intact hypothalamic-pituitary-gonadal axis. In addition to improving serum testosterone levels, it has been shown to improve hypogonadal symptoms ^21, 28^ and improve bone mineral density while avoiding adverse effects on PSA and hematocrit and gynecomastia ^29^ and may be more economical than testosterone therapy ^30^. Though it seems a viable alternative to testosterone, it is not FDA approved for use in the treatment of hypogonadism. Potential drawbacks include an increased risk of thromboembolism as seen with other selective estrogen-receptor modulators, such as raloxifene, and a diminution of estrogen effects in the male, including effects on libido and bone mineral density.

Enclomiphene is the *trans*-isomer of clomiphene. It is being studied as a treatment for hypogonadism in men with intact pituitary who wish to retain spermatogenesis during treatment. Several studies support the contention that spermatogenesis is better preserved with this approach ^31, 32^, but limited data on fertility preservation are available. As a result, this medication has not yet been approved for clinical use but is an interesting compound for future study in men who wish to maintain sperm production during treatment for hypogonadism.

### Aromatase inhibitors

Aromatase converts testosterone to E2 in various tissues, particularly fat. Aromatase inhibitors prevent this and lower E2 ^33^, which in turn prevents feedback inhibition of GnRH and causes increased release, thereby raising serum testosterone concentrations as well as intra-testicular testosterone. Agents such as letrozole and anastrozole have been investigated for the treatment of male hypogonadism. These have uniformly shown an increase in serum testosterone concentrations ^33– 35^ and some have reported improvement in sexual desire ^34^, lean mass, muscle strength, and physical function ^36^. The other advantages of aromatase inhibitors over testosterone therapy are that the lower estrogen state does not increase prostate volume or lower urinary tract symptoms (LUTS) ^37^ and it does not lower, or may even improve, sperm parameters ^38^. These factors make them a possible alternative to testosterone in obese hypogonadal men with high E2 concentrations, older hypogonadal men with BPH, and younger hypogonadal and subfertile men. However, the effect of lowering E2 levels can cause adverse effects on bone mineral density ^36, 39, 40^, while other studies have shown no such effects ^41^. Also, recent studies have highlighted the importance of E2 in maintaining sexual desire ^42^ and so lowering E2 concentrations in hypogonadal men may affect symptom improvement significantly.

### Selective androgen receptor modulators

Androgen therapies mostly have beneficial effects on skeletal muscle and bone; however, they tend to cause negative effects on erythrocytes (erythrocytosis), prostate (BPH), hair (alopecia), and skin (acne). Therefore, an ideal therapeutic agent for the treatment of hypogonadism would have tissue-specific effects so that we can achieve the “good” without incurring the “bad”. This is the concept from which the development of selective androgen receptor modulators (SARMs) has stemmed. These agents act as tissue-specific androgen-receptor ligands, mainly acting at the level of skeletal tissue or bone. Studies have looked at their role in the treatment of chronic disease or cancer cachexia, frailty, sarcopenia, osteoporosis, hypogonadism, prostate cancer, and male contraception ^43^, but these are mostly pre-clinical or early-phase clinical studies of short duration. Rodent models have shown anabolic effects on muscle and bone with reduced impact on prostate growth as well as improvement in sexual behavior ^44^. One promising 12-week, double-blinded, placebo-controlled phase 2 trial of GTx-024 (enobosarm) in 120 healthy men older than 60 years of age showed dose-dependent increases in total lean body mass and improvement in physical function and insulin resistance ^45^. However, there is not enough evidence to recommend these as an alternative to testosterone for the treatment of hypogonadism as yet.

## Challenges for alternative testosterone therapies for hypogonadism

While alternatives to testosterone therapy have appeal, particularly for men wishing to avoid suppression of spermatogenesis by testosterone, there is greater uncertainly regarding their risks and benefits, as they have been less well studied. In addition, FDA approval for these therapies has been difficult to obtain, as the FDA has indicated that raising testosterone is not acceptable as a primary endpoint for non-testosterone therapies. Instead, the FDA requires data on improvement of the signs and symptoms of hypogonadism ^46^. Such studies will likely require larger samples and better outcome measures, work that is somewhat hampered by the paucity of validated patient-reported outcome measures in the field.

## Conclusions

Treatment of male hypogonadism remains an area that requires in-depth discussion of the risks and benefits of therapy with the patient before proceeding. With the numerous testosterone formulations that exist and are being developed, patients have a variety of options to choose from, depending on their preferences. Additionally, there are a number of non-testosterone alternative therapies too that can be considered, particularly in men desiring fertility or wishing to avoid specific side effects of testosterone therapy. Still newer therapies, currently in the stages of early clinical trials (selective androgen receptor modulators), may indeed be the future of androgen replacement therapy and hint at the promise of more benefits than harm, which would simplify these patient discussions and decisions.

## Abbreviations

BPH, benign prostatic hyperplasia; DHT, dihydrotestosterone; E2, estradiol; FDA, US Food and Drug Administration; FSH, follicle-stimulating hormone; GnRH, gonadotropin-releasing hormone; hCG, human chorionic gonadotropin; hMG, human menopausal gonadotropin; LH, luteinizing hormone; LUTS, lower urinary tract symptoms; PSA, prostate-specific antigen; REMS, Risk Evaluation and Mitigation Strategy; rhFSH, recombinant human follicle-stimulating hormone; SARMs, selective androgen receptor modulators.

## References

[1] Bhasin S Cunningham GR Hayes FJ: Testosterone therapy in men with androgen deficiency syndromes: an Endocrine Society clinical practice guideline. *J Clin Endocrinol Metab.* 2010;95(6):2536–59. 10.1210/jc.2009-2354 20525905

[2] Harman SM Metter EJ Tobin JD: Longitudinal effects of aging on serum total and free testosterone levels in healthy men. Baltimore Longitudinal Study of Aging. *J Clin Endocrinol Metab.* 2001;86(2):724–31. 10.1210/jcem.86.2.7219 11158037

[3] Feldman HA Longcope C Derby CA: Age trends in the level of serum testosterone and other hormones in middle-aged men: longitudinal results from the Massachusetts male aging study. *J Clin Endocrinol Metab.* 2002;87(3):589–98. 10.1210/jcem.87.2.8201 11836290

[4] Tan RS Salazar JA: Risks of testosterone replacement therapy in ageing men. *Expert Opin Drug Saf.* 2004;3(6):599–606. 10.1517/14740338.3.6.599 15500418

[5] Contraceptive efficacy of testosterone-induced azoospermia in normal men. World Health Organization Task Force on methods for the regulation of male fertility. *Lancet.* 1990;336(8721):955–9. 1977002

[6] Goodman N Guay A Dandona P: American Association of Clinical Endocrinologists and American College of Endocrinology position statement On The Association Of Testosterone And Cardiovascular Risk. *Endocr Pract.* 2015;21(9):1066–73. 10.4158/EP14434.PS 26355962

[7] Morgentaler A Miner MM Caliber M: Testosterone therapy and cardiovascular risk: advances and controversies. *Mayo Clin Proc.* 2015;90(2):224–51. 10.1016/j.mayocp.2014.10.011 25636998

[8] Snyder PJ Lawrence DA: Treatment of male hypogonadism with testosterone enanthate. *J Clin Endocrinol Metab.* 1980;51(6):1335–9. 10.1210/jcem-51-6-1335 6777395

[9] Sih R Morley JE Kaiser FE: Testosterone replacement in older hypogonadal men: a 12-month randomized controlled trial. *J Clin Endocrinol Metab.* 1997;82(6):1661–7. 10.1210/jcem.82.6.3988 9177359

[10] Schürmeyer T Nieschlag E: Comparative pharmacokinetics of testosterone enanthate and testosterone cyclohexanecarboxylate as assessed by serum and salivary testosterone levels in normal men. *Int J Androl.* 1984;7(3):181–7. 10.1111/j.1365-2605.1984.tb00775.x 6434435

[11] Swerdloff RS Wang C Cunningham G: Long-term pharmacokinetics of transdermal testosterone gel in hypogonadal men. *J Clin Endocrinol Metab.* 2000;85(12):4500–10. 10.1210/jcem.85.12.7045 11134099

[12] McCullough AR Khera M Goldstein I: A multi-institutional observational study of testosterone levels after testosterone pellet (Testopel(®)) insertion. *J Sex Med.* 2012;9(2):594–601. 10.1111/j.1743-6109.2011.02570.x 22240203

[13] Rogol AD Tkachenko N Bryson N: Natesto ^TM^, a novel testosterone nasal gel, normalizes androgen levels in hypogonadal men. *Andrology.* 2016;4(1):46–54. 10.1111/andr.12137 26695758

[14] Wang C Swerdloff R Kipnes M: New testosterone buccal system (Striant) delivers physiological testosterone levels: pharmacokinetics study in hypogonadal men. *J Clin Endocrinol Metab.* 2004;89(8):3821–9. 10.1210/jc.2003-031866 15292312

[15] Yin AY Htun M Swerdloff RS: Reexamination of pharmacokinetics of oral testosterone undecanoate in hypogonadal men with a new self-emulsifying formulation. *J Androl.* 2012;33(2):190–201. 10.2164/jandrol.111.013169 21474786 PMC4168025

[16] Kühnert B Byrne M Simoni M: Testosterone substitution with a new transdermal, hydroalcoholic gel applied to scrotal or non-scrotal skin: a multicentre trial. *Eur J Endocrinol.* 2005;153(2):317–26. 10.1530/eje.1.01964 16061839

[17] Wittert GA Harrison RW Buckley MJ: An open-label, phase 2, single centre, randomized, crossover design bioequivalence study of AndroForte 5 testosterone cream and Testogel 1% testosterone gel in hypogonadal men: study LP101. *Andrology.* 2016;4(1):41–5. 10.1111/andr.12129 26754331

[18] Page ST Lin DW Mostaghel EA: Dihydrotestosterone administration does not increase intraprostatic androgen concentrations or alter prostate androgen action in healthy men: a randomized-controlled trial. *J Clin Endocrinol Metab.* 2011;96(2):430–7. 10.1210/jc.2010-1865 21177791 PMC3048323

[19] Aydogdu A Swerdloff RS: Emerging medication for the treatment of male hypogonadism. *Expert Opin Emerg Drugs.* 2016;21(3):255–66. 10.1080/14728214.2016.1226799 27552127

[20] Ly LP Jimenez M Zhuang TN: A double-blind, placebo-controlled, randomized clinical trial of transdermal dihydrotestosterone gel on muscular strength, mobility, and quality of life in older men with partial androgen deficiency. *J Clin Endocrinol Metab.* 2001;86(9):4078–88. 10.1210/jcem.86.9.7821 11549629

[21] Santen RJ Leonard JM Sherins RJ: Short- and long-term effects of clomiphene citrate on the pituitary-testicular axis. *J Clin Endocrinol Metab.* 1971;33(6):970–9. 10.1210/jcem-33-6-970 5135636

[22] George B Bantwal G: Endocrine management of male subfertility. *Indian J Endocrinol Metab.* 2013;17(Suppl 1):S32–4. 10.4103/2230-8210.119500 24251201 PMC3830347

[23] Young J Couzinet B Chanson P: Effects of human recombinant luteinizing hormone and follicle-stimulating hormone in patients with acquired hypogonadotropic hypogonadism: study of Sertoli and Leydig cell secretions and interactions. *J Clin Endocrinol Metab.* 2000;85(9):3239–44. 10.1210/jcem.85.9.6811 10999815

[24] Liu PY Wishart SM Handelsman DJ: A double-blind, placebo-controlled, randomized clinical trial of recombinant human chorionic gonadotropin on muscle strength and physical function and activity in older men with partial age-related androgen deficiency. *J Clin Endocrinol Metab.* 2002;87(7):3125–35. 10.1210/jcem.87.7.8630 12107212

[25] Meier C Liu PY Ly LP: Recombinant human chorionic gonadotropin but not dihydrotestosterone alone stimulates osteoblastic collagen synthesis in older men with partial age-related androgen deficiency. *J Clin Endocrinol Metab.* 2004;89(6):3033–41. 10.1210/jc.2003-031992 15181095

[26] Burris AS Rodbard HW Winters SJ: Gonadotropin therapy in men with isolated hypogonadotropic hypogonadism: the response to human chorionic gonadotropin is predicted by initial testicular size. *J Clin Endocrinol Metab.* 1988;66(6):1144–51. 10.1210/jcem-66-6-1144 3372679

[27] Maggi M Buvat J Corona G: Hormonal causes of male sexual dysfunctions and their management (hyperprolactinemia, thyroid disorders, GH disorders, and DHEA). *J Sex Med.* 2013;10(3):661–77. 10.1111/j.1743-6109.2012.02735.x 22524444

[28] Shabsigh A Kang Y Shabsign R: Clomiphene citrate effects on testosterone/estrogen ratio in male hypogonadism. *J Sex Med.* 2005;2(5):716–21. 10.1111/j.1743-6109.2005.00075.x 16422830

[29] Moskovic DJ Katz DJ Akhavan A: Clomiphene citrate is safe and effective for long-term management of hypogonadism. *BJU Int.* 2012;110(10):1524–8. 10.1111/j.1464-410X.2012.10968.x 22458540

[30] Taylor F Levine L: Clomiphene citrate and testosterone gel replacement therapy for male hypogonadism: efficacy and treatment cost. *J Sex Med.* 2010;7(1 Pt 1):269–76. 10.1111/j.1743-6109.2009.01454.x 19694928

[31] Wiehle RD Fontenot GK Wike J: Enclomiphene citrate stimulates testosterone production while preventing oligospermia: a randomized phase II clinical trial comparing topical testosterone. *Fertil Steril.* 2014;102(3):720–7. 10.1016/j.fertnstert.2014.06.004 25044085

[32] Kim ED McCullough A Kaminetsky J: Oral enclomiphene citrate raises testosterone and preserves sperm counts in obese hypogonadal men, unlike topical testosterone: restoration instead of replacement. *BJU Int.* 2016;117(4):677–85. 10.1111/bju.13337 26496621

[33] Loves S Ruinemans-Koerts J Boer H de: Letrozole once a week normalizes serum testosterone in obesity-related male hypogonadism. *Eur J Endocrinol.* 2008;158(5):741–7. 10.1530/EJE-07-0663 18426834

[34] Richardson D Goldmeier D Frize G: Letrozole versus testosterone. a single-center pilot study of HIV-infected men who have sex with men on highly active anti-retroviral therapy (HAART) with hypoactive sexual desire disorder and raised estradiol levels. *J Sex Med.* 2007;4(2):502–8. 10.1111/j.1743-6109.2007.00451.x 17367446

[35] Leder BZ Finkelstein JS: Effect of aromatase inhibition on bone metabolism in elderly hypogonadal men. *Osteoporos Int.* 2005;16(12):1487–94. 10.1007/s00198-005-1890-8 15856361

[36] Dias JP Melvin D Simonsick EM: Effects of aromatase inhibition vs. testosterone in older men with low testosterone: randomized-controlled trial. *Andrology.* 2016;4(1):33–40. 10.1111/andr.12126 26588809 PMC5836312

[37] Dias JP Melvin D Shardell M: Effects of Transdermal Testosterone Gel or an Aromatase Inhibitor on Prostate Volume in Older Men. *J Clin Endocrinol Metab.* 2016;101(4):1865–71. 10.1210/jc.2016-1111 26950683 PMC4880169

[38] Ribeiro MA Gameiro LF Scarano WR: Aromatase inhibitors in the treatment of oligozoospermic or azoospermic men: a systematic review of randomized controlled trials. *JBRA Assist Reprod.* 2016;20(2):82–8. 10.5935/1518-0557.20160019 27244767

[39] Burnett-Bowie SM McKay EA Lee H: Effects of aromatase inhibition on bone mineral density and bone turnover in older men with low testosterone levels. *J Clin Endocrinol Metab.* 2009;94(12):4785–92. 10.1210/jc.2009-0739 19820017 PMC2795655

[40] Hero M Toiviainen-Salo S Wickman S: Vertebral morphology in aromatase inhibitor-treated males with idiopathic short stature or constitutional delay of puberty. *J Bone Miner Res.* 2010;25(7):1536–43. 10.1002/jbmr.56 20200972

[41] Eastell R Adams JE Coleman RE: Effect of anastrozole on bone mineral density: 5-year results from the anastrozole, tamoxifen, alone or in combination trial 18233230. *J Clin Oncol.* 2008;26(7):1051–7. 10.1200/JCO.2007.11.0726 18309940

[42] Finkelstein JS Yu EW Burnett-Bowie SM: Gonadal steroids and body composition, strength, and sexual function in men. *N Engl J Med.* 2013;369(25):2455–2457. 10.1056/NEJMc1313169 24350954

[43] Zhang X Sui Z: Deciphering the selective androgen receptor modulators paradigm. *Expert Opin Drug Discov.* 2013;8(2):191–218. 10.1517/17460441.2013.741582 23231475

[44] Miner JN Chang W Chapman MS: An orally active selective androgen receptor modulator is efficacious on bone, muscle, and sex function with reduced impact on prostate. *Endocrinology.* 2007;148(1):363–73. 10.1210/en.2006-0793 17023534

[45] Dalton JT Barnette KG Bohl CE: The selective androgen receptor modulator GTx-024 (enobosarm) improves lean body mass and physical function in healthy elderly men and postmenopausal women: results of a double-blind, placebo-controlled phase II trial. *J Cachexia Sarcopenia Muscle.* 2011;2(3):153–61. 10.1007/s13539-011-0034-6 22031847 PMC3177038

[46] FDA Briefing Document. Bone, Reproductive and Urologic Drugs Advisory Committee (BRUDAC) December 6, 2016. Reference Source

